# Treatment of interprosthetic femoral fractures with an interposition prosthesis: A technical note

**DOI:** 10.3109/17453674.2013.795434

**Published:** 2013-05-31

**Authors:** Mustafa Citak, Till Orla Klatte, Daniel Kendoff, Carl Haasper, Thorsten Gehrke, Matthias Gebauer

**Affiliations:** ^1^Department of Orthopaedic Surgery, Helios ENDO Klinik Hamburg; ^2^Department of Trauma-, Hand- and Reconstructive Surgery, University Medical Center Hamburg-Eppendorf, Hamburg, Germany.

Treatment of interprosthetic femoral fractures following ipsilateral total hip and hinged knee arthroplasty is challenging, particularly when there is severe bone loss. When open reduction with internal plate fixation is not possible, a total femur replacement may be considered (Friesecke et al. 2005, Peters et al. 2006). Here we describe a new surgical technique for the treatment of these fractures using an interposition prosthesis and we present our results from 4 cases.

## Surgical technique

With the patient in the lateral position, a lateral approach was used for exposure of the fracture. Extensive debridement included removal of cement and comminuted bone around the femoral stem tip, the interprosthetic region, and the knee femoral component. Skeletonizing of the hip and knee stems in a length of about 8 cm was then performed. After validation of stable implants in these areas, the 2 sleeves of the custom-made prosthesis were temporarily put onto the exposed stems and were centered using the set screws. After adjusting the correct restoration of the femoral rotation and length, the 2 bushings were temporarily tightened with 2 fixation screws.

**Figure 1.**
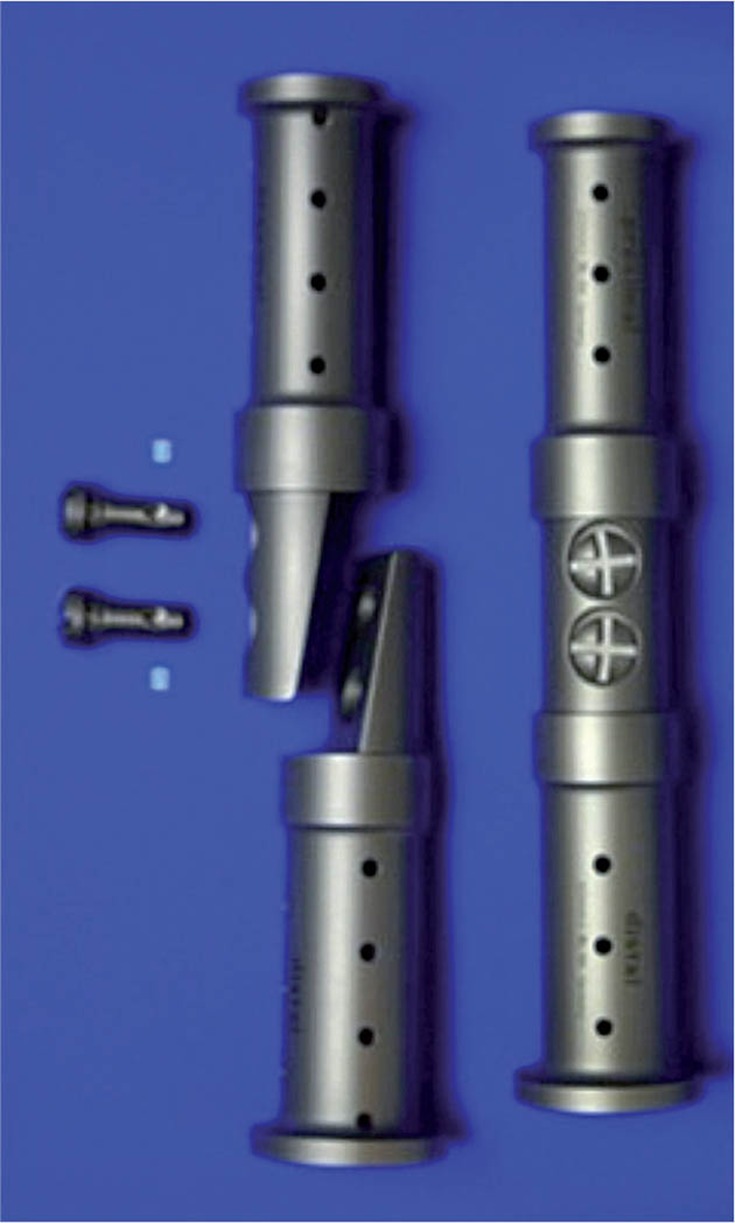
The custom-made interposition device consisting of 2 coupling parts. The 2 parts are fixed using 2 screws.

After achieving a solid bridging of the former interprosthetic fracture zone and reconstruction of femur length, the proximal and lower parts of the prosthesis were again loosened before final implantation. After extensive jet lavage, the proximal and distal sleeves were cemented using a two-step technique. After cement hardening, the coupling mechanism was fixed using 2 screws (Figure 1). Full weight bearing was allowed immediately in all patients.

## Patients

**Figure 2.**
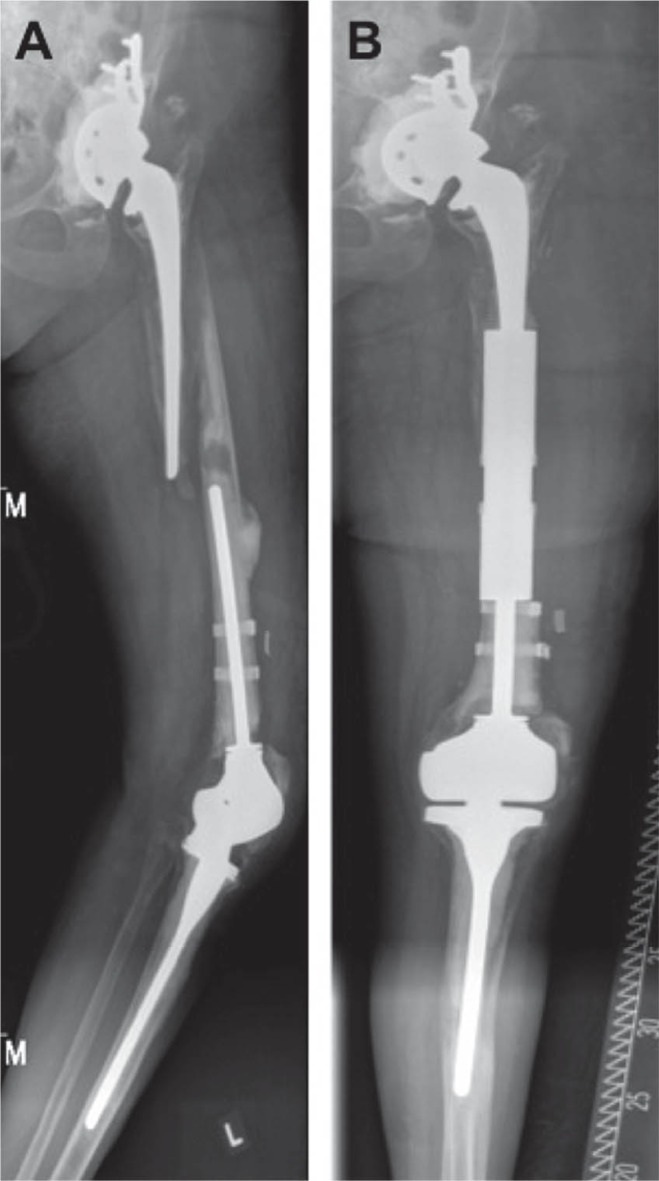
A. Interprosthetic femoral fracture in a 78-year-old woman. B. Follow-up radiograph after 1.5 years. At this stage the patient was mobile with a walker and free of pain (HSS 55).

Between January 1996 and February 2012, we treated 4 patients with interprosthetic femoral fractures (3 of them women) (Figure 2) using a custom-made interposition device (Waldemar Link GmbH, Hamburg, Germany) (Figure 1). Mean age was 74 (59–86) years. The fractures occurred mean 18 (13–28) years after primary THA and mean 14 (10–17) years after primary TKA. At the latest follow-up, after mean 8 (0.5–16) years, revision surgery with a total femur replacement was required in 1 case due to aseptic loosening. No other complications requiring revision surgery occurred.

## Discussion

Michla et al. (2010) described the complex problem of interprosthetic femoral fractures in elderly patients and pointed out that this type of injury will become more common. Mamczak et al. (2010) and Sah et al. (2010) presented successful results of open reduction and internal plate fixation (LISS, LCP) in the treatment of interprosthetic fractures in a total of 48 fractures; all of them had a surface knee arthroplasty.

Treatment options for interprosthetic femoral fractures following total hip and stemmed knee arthroplasty are limited, particularly when there is severe bone loss in elderly patients. Kenny et al. (1998) presented 4 such cases treated with internal fixation devices, and all of them failed. These authors concluded that surface knee arthroplasty provides a better opportunity for internal fixation than a knee arthroplasty with a stemmed femoral component.

Here we have described a new surgical technique with a custom-made interposition device for treatment of these specific fractures. The characteristics of this interposition device have not been published before. The explicit indication for this device are interprosthetic fractures of the femur with coexisting stemmed hip and stemmed femoral knee components.

Due to our encouraging results with this interposition device in 4 cases, we believe that it is a valuable treatment option for interprosthetic femoral fractures after ipsilateral hip and stemmed knee joint replacement.
